# Protective Effect of *Rhus chinensis* Mill. Fruits on 3,5-Diethoxycarbonyl-1,4-Dihydrocollidine-Induced Cholestasis in Mice via Ameliorating Oxidative Stress and Inflammation

**DOI:** 10.3390/nu14194090

**Published:** 2022-10-01

**Authors:** Yilin Sun, Shengbao Cai, Yuanyue Zhang, Nan Ma, Junjie Yi, Xiaosong Hu, Tao Wang

**Affiliations:** 1 Faculty of Food Science and Engineering, Kunming University of Science and Technology, Kunming 650500, China; 2 Key Laboratory of Dairy Science, Ministry of Education, Department of Food Science, Northeast Agricultural University, Harbin 150030, China; 3 College of Food Science and Nutritional Engineering, China Agricultural University, Beijing 100083, China

**Keywords:** cholestasis, *Rhus chinensis* Mill., polyphenols, liver damage, oxidative stress, inflammation

## Abstract

This study focused on the preventive effects of the extracts of *Rhus chinensis* Mill. (RCM) fruits on cholestasis induced by 3,5-diethoxycarbonyl-1,4-dihydrocollidine (DDC) in mice. The results showed that RCM extracts could significantly ameliorate DDC-induced cholestasis via multiple mechanisms, including (1) alleviating liver damage via enhancing antioxidant capacity, such as increasing the contents of glutathione, superoxide dismutase, and catalase and inhibiting the levels of malondialdehyde; (2) preventing liver inflammation by suppressing NF-κB pathway and reducing proinflammatory cytokines secretion (e.g., tumor necrosis factor-α, interleukin-1β, and interleukin-6); (3) inhibiting liver fibrosis and collagen deposition by regulating the expression of transforming growth factor-β and α-smooth muscle actin; (4) modulating abnormal bile acid metabolism through increasing the expression of bile salt export pump and multidrug resistance-associated protein 2. This study was the first to elucidate the potential preventive effect of RCM extracts on DDC-induced cholestasis in mice from multiple pathways, which suggested that RCM fruits could be considered as a potential dietary supplement to prevent cholestasis.

## 1. Introduction

The liver is a vital organ for bile anabolism with functions of detoxification and bile secretion control, which makes it particularly vulnerable [1]. Cholestasis is a common liver disease in the clinic and its pathological manifestations are obstruction of bile flow and excessive accumulation of bile components [2,3]. Oxidative stress, inflammation, and transporter disorders are considered to be the underlying pathological mechanisms involved in the development of cholestasis [4]. Chronic and persistent cholestasis can eventually lead to liver fibrosis and cirrhosis [5]. Primary sclerosing cholangitis (PSC) and primary biliary cholangitis (PBC) are the two major disease modes of cholestasis, with an estimated 200–500 cases per million inhabitants [6]; 3,5-diethoxycarbonyl 1,4-dihydrocoridine (DDC) is often used to establish animal models of PSC cholestasis. This model recapitulates the major histopathological features of human cholestatic liver disease, such as peritoneal fibrosis and inflammatory cell infiltration [7]. Although ursodeoxycholic acid (UDCA) is commonly used in the treatment of cholestatic liver disease because of its ability to protect hepatocytes and cholangiocytes, the clinical effect of UDCA is not ideal and 40% of patients had no significant curative effect during treatment [8]. Therefore, developing efficient and safe dietary interventions from natural products to prevent cholestasis is necessarily urgent.

In recent decades, several natural plants and their active ingredients have been shown to have preventive and protective effects against cholestatic liver injury, mainly involving the alleviation of bile acid metabolism abnormalities, oxidative stress, inflammatory responses, and fibrosis. In animal models of cholestasis, the levels of oxidative stress markers in the liver were increased, indicating that oxidative stress is one of the triggers of cholestasis [9]. A prior study showed that *Platycodon grandiflorum* could attenuate DDC-induced cholestasis via reducing the level of malondialdehyde (MDA) and raising the activity of superoxide dismutase (SOD) [10]. More seriously, oxidative stress can further activate inflammatory pathways, thereby promoting the synthesis and secretion of inflammatory cytokines [11,12]. Oxidative stress and inflammation can both activate hepatic stellate cells (HSCs), which then accelerates liver fibrosis. Transforming growth factor-beta (TGF-β) and α-smooth muscle actin (α-SMA) are crucial indicators of fibrosis [13]. Wang et al. [14] found that *Glechoma hederacea* could reduce inflammation by down-regulating the expression of NF-κB and reducing the expression of α-SMA and TGF-β to inhibit fibrosis caused by cholestasis. Moreover, apigenin can inhibit DDC-induced cholestasis and fibrosis by reducing inflammation, and down-regulating the expression of TGF-β and α-SMA [15]. Bile duct transporters also play an important role in bile metabolism, and the expression of bile salt export pump (BSEP) and multidrug resistance-associated protein2 (MRP2) is closely related to the tubular transport of bile acids [16]. Treatment with *Abelmoschus Manihot* (L.) Medic could alleviate cholestasis by increasing the expression of BSEP and MRP2 [17]. Compared with traditional clinical hepatoprotective drugs, natural products have been widely studied and utilized due to their advantages of fewer side effects and higher safety, which can be used as adjuvant therapy to provide theoretical guidance for clinical prevention and treatment of related diseases [18].

*Rhus chinensis* Mill. (RCM), a member of the genus *Rhus*, is widely cultivated throughout the world. The official name of *R. chinensis* in Chinese was “Yan Fu Mu”, and its fruits were named “Yan Fu Zi”, herein “Yan” means salt in Chinese, which refers to that the plant can produce salt [19]. The ripe fruit of RCM is nutritious and eaten as a condiment in many areas [20]. According to folk pharmacopeia, RCM has medicinal value and its fruit can be used as a treatment for jaundice and hepatitis [21]. Our previous studies have shown that the extracts of RCM are content with a variety of polyphenolic flavonoids, such as gallic acid and quercetin-3-*O*-rhamnoside, which have been shown to have a good antioxidant capacity in vitro [22]. Furthermore, the ethanol extracts of RCM could alleviate acetaminophen-induced liver injury by inhibiting oxidation and inflammation [23]. Therefore, RCM has great potential in preventing liver-related diseases because of its potential antioxidant and anti-inflammatory effects. Since cholestatic liver injury is partly related to oxidative stress and inflammatory responses, it can be speculated that RCM may also have great potential in alleviating cholestatic liver injury. To further illustrate this hypothesis, this study is the first to investigate the preventive effect of RCM fruits on DDC-induced cholestasis through multiple pathways, which can promote the application of these fruits as functional foods, and provide theoretical research and application guidance for the prevention of cholestasis. However, this study only stays at the animal level, and the specific medicinal value of this fruit on cholestasis needs to be studied in subsequent clinical trials.

## 2. Materials and Methods

### 2.1. Chemical and Reagents

DDC (D154604) was obtained from Aladdin (Shanghai, China). Biochemical kits, including alanine aminotransferase (ALT), aspartate aminotransferase (AST), alkaline phosphatase (ALP), reduced glutathione (GSH), MDA, SOD, and catalase (CAT) were supplied by Nanjing Jiancheng Bioengineering Institute (Nanjing, China). Enzyme-linked immunosorbent assay (ELISA) detection kits, including interleukin-6 (IL-6), tumor necrosis factor-α (TNF-α), and interleukin-1β (IL-1β), were obtained from Multi Sciences Biotech Company (Hangzhou, China). All phenolic standards were purchased from Must bio-technology CO., Ltd. (Chengdu, Sichuan, China) with purity ≥ 97%.

### 2.2. Preparation and Phytochemical Characterization of the Sample

The RCM fruits were purchased from the Kunming Plant Classification Biotechnology Co., Ltd., China, in 2019 and stored at −30 °C. The ethanol extracts of RCM were prepared in line with our previous study [24]. The extracts were characterized by a high-resolution mass spectrometer in the negative mode. The parameters of HPLC were set as follows: mobile phases, 0.1% formic acid in water (A) and acetonitrile (B); flow rate, 0.1 mL/min; elution procedure, 0–2 min, 5% B; 2–4 min, 5–20% B; 4–12 min, 20–30% B; 12–15 min, 30–50% B; 15–18 min, 50–5% B; 18–20 min, 5% B; column temperature, 30 °C; the volume of sample injection, 2.0 μL. The base peak chromatogram and phenolic compounds of ethanol extract of *Rhus chinensis* Mill. fruits are shown in Figure S1 and Table S1, respectively.

### 2.3. Animal Experimental Design

Forty specific pathogen-free male C57 BL/6 J mice (18–20 g) were provided by Hunan SJA Laboratory Animal Co., Ltd. (Hunan, China; Certificate No. SCXK (Xiang) 2021-003, Ethical approval No. PZWH (Dian) K2022-0013). All mice were housed in a prescribed animal feeding environment (22 ± 2 °C, 40–70% humidity, and alternative 12 h light-dark cycles). Animal experiments were carried out strictly following the Guidelines for the Care and Use of Experimental Animals.

**Figure 1:**
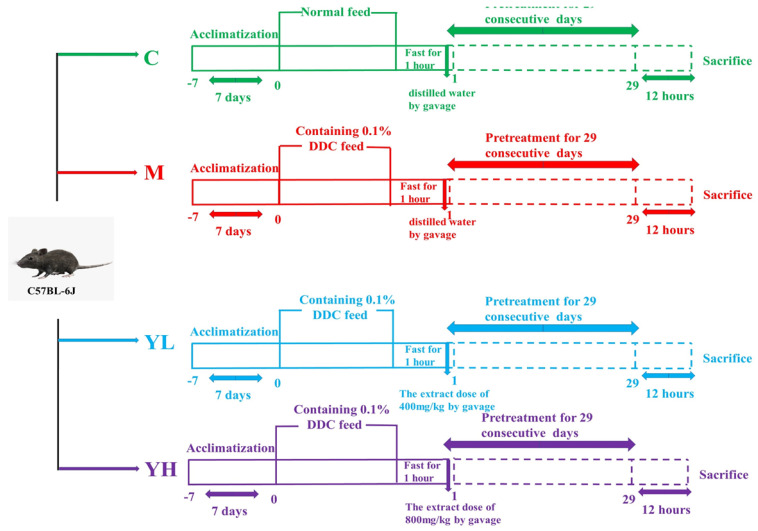
Outline of the experimental design about the effect of the extracts of RCM on DDC−induced cholestasis in mice.

The detailed experimental protocol is shown in Figure 1. Briefly, after 1 week of adaptive feeding, all mice were randomly divided into 4 groups (*n* = 10), including the control group (C), DDC-induced cholestasis model group (M), low-dose “Yan Fu Zi” extracts (400 mg/kg b.w.) treatment group (YL) and high-dose “Yan Fu Zi” extracts (800 mg/kg b.w.) treatment group (YH). The intervention doses used in this study were referenced as previously described [25]. Mice in group C were fed with a normal diet, while mice in groups M, YL, and YH were fed with a model diet containing 0.1% DDC. The normal feed was supplied from Kunming Medical University, and the DDC feed (containing 0.1% DDC) was supplied from Kunming Zanna Biotechnology Co., Ltd. (Kunming, Yunnan, China). The selection of the model diet was based on the previous study [15]. YL and YH groups were gavaged with RCM extracts every day (400 or 800 mg/kg b.w., 10 mL/kg b.w.). While mice in groups C and M were gavaged with the same volume of distilled water. Mice were anesthetized with 2% isoflurane after 30 days of administration. Fresh blood from eyeballs was collected using tubes that contained heparin sodium and centrifuged at 2200× *g* and 4 °C for 5 min. After centrifugation, plasma was taken and preserved at −80 ℃ for further analysis. Liver tissue was weighed and divided into 2 parts: one was immersed in 4% paraformaldehyde solution for histopathological analysis; the other one was stored at −80 °C for biochemical and protein expression analysis. 

### 2.4. Biochemical Analyses

Biochemical indices in plasma (ALT, AST, and ALP) and liver tissues (GSH, SOD, CAT, and MDA) were measured based on corresponding commercial kits (Nanjing Jiancheng Bioengineering Institute). The liver tissue homogenate was prepared as described previously [25].

### 2.5. ELISA Measurements

The contents of IL-6, TNF-α, and IL-1β in the plasma of mice were tested via corresponding ELISA kits following the manufacturer’s protocol.

### 2.6. Histopathological Assessments

Hepatic lobules of each liver were immersed in 4% paraformaldehyde, dehydrated in gradient alcohol, embedded in paraffin, sliced, dewaxed, and rehydrated. Finally, each section was stained with hematoxylin-eosin staining (H&E), Masson staining, and Sirius red staining and then observed under the Olympus IX83 microscope (Tokyo, Japan).

### 2.7. Western Blot Analyses 

The expressions of NF-κB, IκBα, BSEP, and MRP2 in liver tissue were measured by western blot as our previously described methods [23]. Antibodies, including *p*-IκBα and *p*-NF-κB, were obtained from Affinity (Jiangsu, China), and NF-κB and IκBα were obtained from ABclonal (Wuhan, China). BSEP was obtained from Neobioscience (Shenzhen, China). MRP2 was purchased from Bioss (Beijing, China).

### 2.8. Immunohistochemical (IHC) Analyses

The preparation of liver slices was conducted according to our previous study [25]. Sections were dewaxed, rehydrated, and blocked with 5% BSA for 30 min. Then, the slice was incubated overnight at 4 °C with TGF-β and α-SMA antibodies. The following steps were consistent with the previously described [24]. 

### 2.9. Statistical Analysis

All results were presented as mean ± standard error (SE). Origin 8.5 software (Northampton, MA, USA) was used to conduct one-way ANOVA, followed by Tukey’s test for multiple comparisons. Relative quantification of IHC staining and western blot results was performed using Image-Pro Plus 6.0 software of Media Cybernetics Inc. (Rockville, MD, USA).

## 3. Results

### 3.1. RCM Alleviated DDC-Induced Liver Injury 

**Figure 2:**
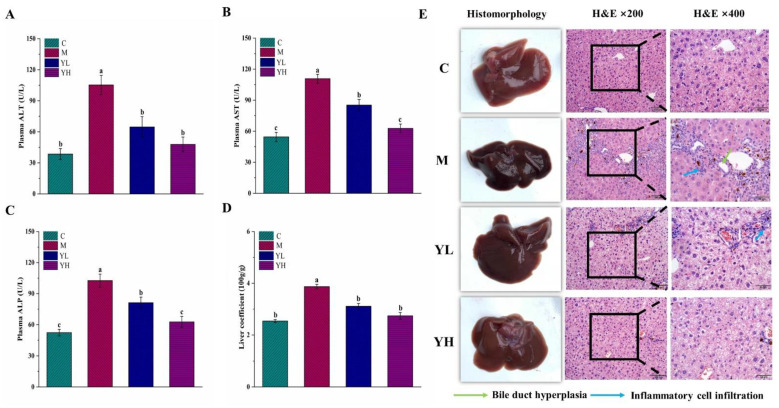
Biochemical results of plasma and histological images in DDC-induced cholestasis in mice. ALT (**A**), AST (**B**), ALP (**C**), liver coefficient (**D**), and liver histomorphology images and H&E staining (×200 and ×400) (**E**). Data are expressed as mean ± SE (*n* = 10). Liver coefficient = [liver weight (g)/body weight (g)] × 100. Mean values with different letters (a, b, and c) are significantly different (*p* < 0.05). C, the control group; M, the model group; YL, low dose of the RCM extracts treatment group (400 mg/kg b.w.); YH, high dose of the RCM extracts treatment group (800 mg/kg b.w.).

The levels of ALT and AST in plasma were presented in Figure 2A,B. Compared with group C, both ALT and AST were dramatically increased in the DDC-induced cholestasis group (*p* < 0.05), while they were reversed in the RCM extracts treatment groups (*p* < 0.05). The amelioration effects of YH were better than that of YL because the levels of these two enzymes were not statistically different between the YH and C groups (*p* > 0.05), but only the ALT level was not statistically different between the YL and C groups (*p* > 0.05). The content of ALP in the DDC-induced cholestasis mice was markedly raised relative to group C (*p* < 0.05), while it was prevented by treatment with RCM extracts (*p* < 0.05), especially in group YH (Figure 2C). The liver coefficient was significantly increased in group M compared with that in group C and decreased in RCM extracts-treated groups (*p* < 0.05, Figure 2D).

The results of histomorphology and H&E analyses of liver tissue are shown in Figure 2E. In group C, the liver showed a normal shape with smooth, dark red, and shiny color and elastic texture. However, the liver color of group M was black with no elasticity. The liver in the RCM extracts-treated groups (especially in group YH) was improved, as evidenced by the color and surface gradually restored, and the morphology was almost no different from that in group C. The results of H&E staining were photographed and observed with a microscope at ×200 and ×400. In group C, the structure of the whole hepatic lobule was normal, the hepatocytes were arranged neatly without obvious necrosis, and the perisinusoidal space was radially distributed. In group M, however, biliary hyperplasia and massive inflammatory cell infiltration were observed around the central vein, and the structure of the perisinusoidal space was also found to be disarranged and almost disappeared. There was a great improvement compared with group M with almost no necrotic area and inflammatory cell infiltration in the RCM extracts treatment groups, particularly in the YH group.

### 3.2. RCM Reduced Oxidative Stress in DDC-Induced Cholestasis

**Figure 3:**
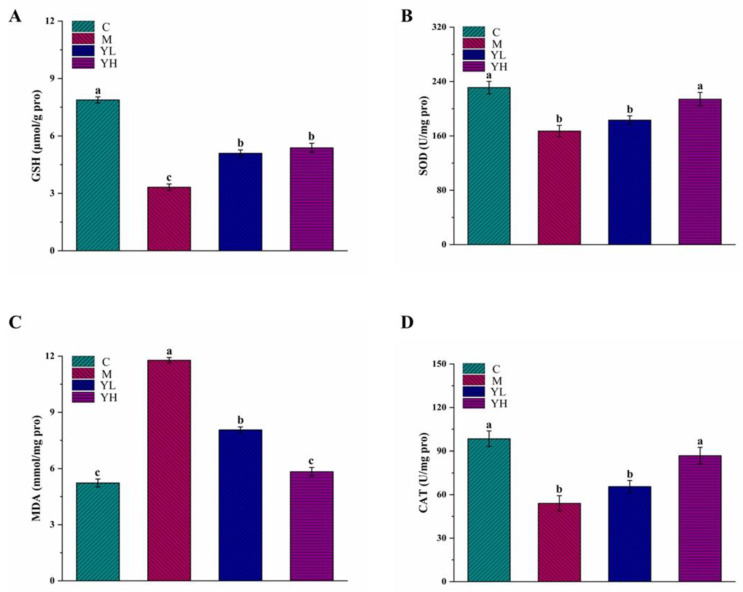
Biochemical results of liver tissue in DDC-induced cholestasis in mice. GSH (**A**), SOD (**B**), MDA (**C**), and CAT (**D**). All data are expressed as mean ± SE (*n* = 10). Mean values with different letters (a, b, and c) are significantly different (*p* < 0.05). C, the control group; M, the model group; YL, low dose of the RCM extracts treatment group (400 mg/kg b.w.); YH, high dose of the RCM extracts treatment group (800 mg/kg b.w.).

The results of GSH and SOD in liver tissue are presented in Figure 3A,B. The levels of GSH and SOD in group M were dramatically decreased relative to group C (*p* < 0.05). After treatment with RCM extracts, the level of GSH was significantly improved in the treatment with RCM extracts groups (*p* < 0.05), but there was still a statistical difference with group C (*p* < 0.05). The SOD level in the YH group was markedly increased relative to group M (*p* < 0.05) and even similar to group C (*p* > 0.05), but there was no statistically significant difference between groups YL and M (*p* > 0.05). The level of MDA in group M was dramatically elevated relative to group C (*p* < 0.05), while it was significantly reversed in the RCM extracts-treated mice, especially in group YH (*p* < 0.05, Figure 3C). The CAT activity in group M was dramatically reduced relative to group C (*p* < 0.05, Figure 3D). After administration of the high-dose of the extracts of RCM fruits (group YH), the activity of CAT was increased (*p* < 0.05), and was found not markedly difference between C and YH groups (*p* > 0.05). Nevertheless, the CAT activity after a low-dose of RCM extracts administration was not markedly raised relative to group M (*p* > 0.05).

### 3.3. RCM Inhibited Liver Inflammation in DDC-Induced Cholestasis 

**Figure 4:**
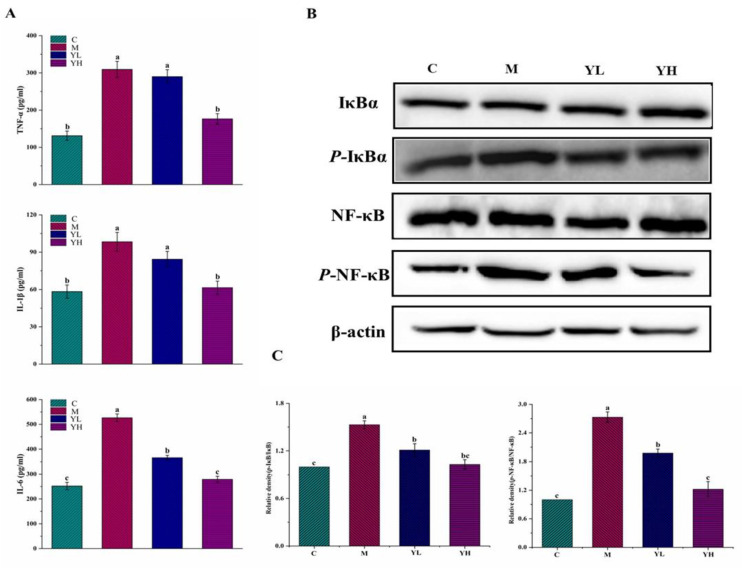
Effects of the extracts of RCM on liver inflammation in DDC-induced cholestasis in mice. The ELISA results of TNF-α, IL-6, and IL-1β (**A**); Western blot results of *p*-IκBα/IκBα and *p*-NF-κB/NF-κB (**B**); the relative expressions of the above proteins were quantified by normalized with group C and β-actin according to grayscale (**C**). Values are expressed as mean ± SE (*n* = 6). Different letters (a, b, and c) indicated significant differences (*p* < 0.05). C, the control group; M, the model group; YL, low dose of the RCM extracts treatment group (400 mg/kg b.w.); YH, high dose of the RCM extracts treatment group (800 mg/kg b.w.).

As shown in Figure 4, compared with group C, DDC administration caused significant increases in levels of TNF-α, IL-6, and IL-1β (*p* < 0.05), while these alterations were obviously prevented by treatment with high-dose RCM extracts (*p* < 0.05), and even arrived no significant difference between groups YH and C (*p* > 0.05). In contrast, the amelioration effects of low-dose RCM extracts were lower than those of high-dose RCM extracts. Only the level of IL-6 in group YL was dramatically decreased in comparison with group M (*p* < 0.05).

The expressions of inflammation-related proteins (e.g., NF-κB, *p*-NF-κB, IκB, and *p*-IκB) were detected by western blot, and the results are presented in Figure 4B,C. The levels of *p*-NF-κB/NF-κB and *p*-IκB/IκB were remarkably upregulated in group M compared with those in group C (*p* < 0.05). However, these abnormal expressions were all markedly alleviated in RCM extracts-treated mice (*p* < 0.05), particularly in group YH.

### 3.4. RCM Ameliorated Liver Fibrosis in DDC-Induced Cholestasis

**Figure 5:**
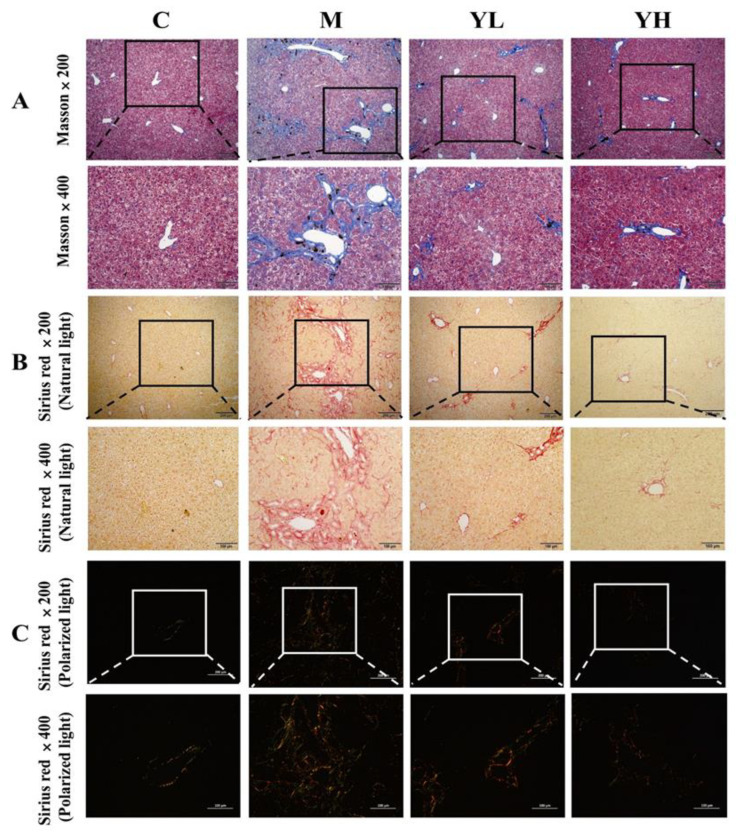

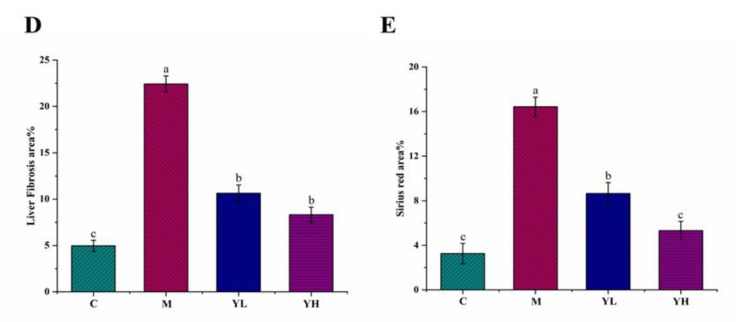
Effects of the extracts of RCM on the formation of collagen fibrosis in DDC-induced cholestasis in mice. Masson staining (**A**), Sirius red staining (Nature light) (**B**), Sirius red staining (Polarized light) (**C**), the liver fibrosis area % (**D**), and Sirius red area % (**E**). Values are expressed as mean ± SE (*n* = 6). Mean values with different letters (a, b, and c) are significantly different (*p* < 0.05). All images were observed by an Olympus IX83 microscope at magnifications ×200, and ×400. Different letters of the same protein indicated significant differences (*p* < 0.05). C, the control group; M, the model group; YL, low dose of the RCM extracts treatment group (400 mg/kg b.w.); YH, high dose of the RCM extracts treatment group (800 mg/kg b.w.).

Results showed that long-term feeding of a diet containing DDC caused serious liver fibrosis in mice (Figure 5). The extent of liver fibrosis was reflected by histopathological analysis, including Masson staining (Figure 5A, ×200 and ×400) and Sirius red staining (Figure 5B,C, ×200 and ×400). Masson staining is a specific staining method to determine collagen deposition, a key method for assessing liver fibrosis. The results in Figure 5A showed that there was almost no collagen deposition in group C, but significant collagen clumps appeared in group M (blue area) (*p* < 0.05), indicating that DDC induced severe liver fibrosis. The RCM extracts treatment groups (YL and YH) significantly prevented collagen formation and improved liver fibrosis (*p* < 0.05). Sirius red staining reacts with collagen fibers to test collagen deposition. The images were taken under natural light (Figure 5B) and polarized light (Figure 5C) to distinguish the type of collagen: type I collagen is observed in yellow light and type II collagen is observed in green [25]. As shown in Figure 5B, no significant collagen was observed in group C under natural light and only a small amount of collagen was found under polarized light (Figure 5C). However, DDC administration caused a significant increase in collagen content (red area, Figure 5B) and both type I and type II collagen increased under polarized light (yellow and green, Figure 5C), indicating that DDC induced severe liver fibrosis. However, the collagen content of the liver was remarkably decreased in the RCM extracts-treated groups. 

**Figure 6:**
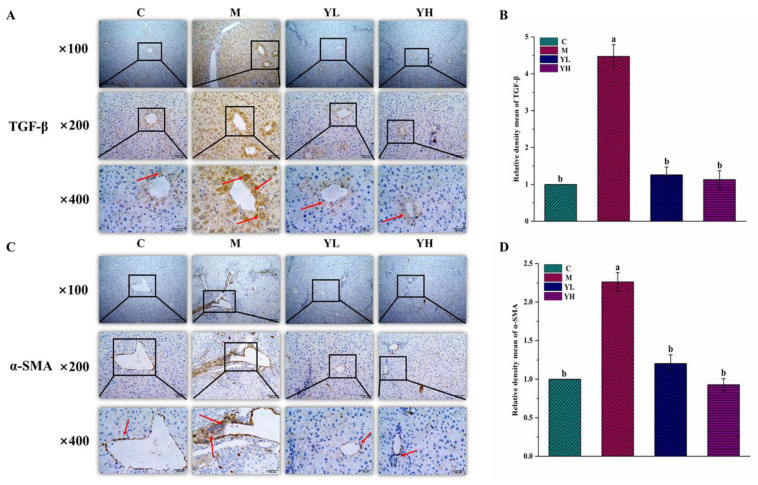
Effects of the RCM extracts on the expressions of TGF-β and α-SMA in DDC-induced cholestasis in mice. IHC of TGF-β expression (**A**), the relative quantification of TGF-β by IHC (**B**), IHC of α-SMA expression (**C**), and the relative quantification of α-SMA by IHC (**D**). Values are expressed as mean ± SE (*n* = 6). Mean values with different letters (a, b, and c) are significantly different (*p* < 0.05). C, the control group; M, the model group; YL, low dose of the RCM extracts treatment group (400 mg/kg b.w.); YH, high dose of the RCM extracts treatment group (800 mg/kg b.w.).

The results of immunohistochemical staining exhibited that the expressions of TGF-β and α-SMA were significantly increased in group M as compared with group C (*p* < 0.05, Figure 6). However, their expressions were significantly down-regulated in both YL and YH treatment groups, especially in YH group (Figure 6).

### 3.5. RCM Improved Bile Duct Proliferation in DDC-Induced Cholestasis

**Figure 7:**
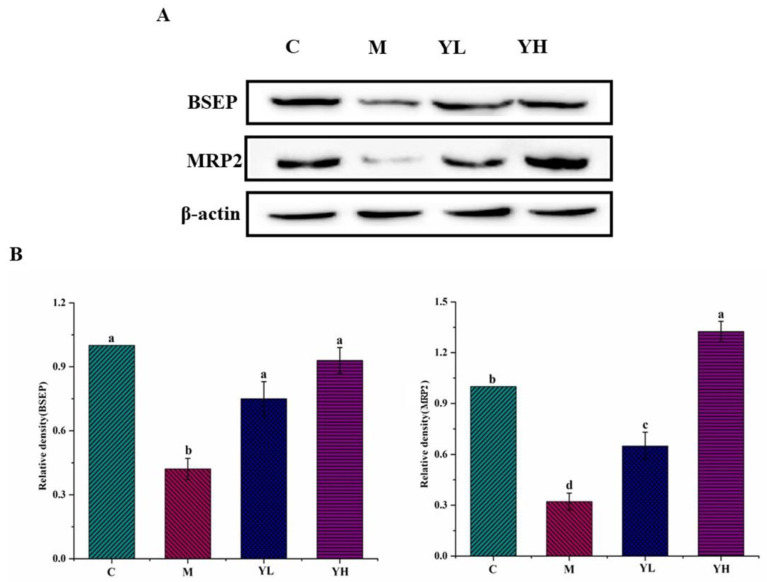
Effects of the RCM extracts on the expression of bile acid transport-related proteins in DDC-induced cholestasis in mice. Western blot analysis of BSEP and MRP2 (**A**), the relative expressions of the above proteins was quantified by normalized with group C and β-actin according to grayscale (**B**). Values are expressed as mean ± SE (*n* = 6). Mean values with different letters (a, b, and c) indicated significant differences (*p* < 0.05). C, the control group; M, the model group; YL, low dose of the RCM extracts treatment group (400 mg/kg b.w.); YH, high dose of the RCM extracts treatment group (800 mg/kg b.w.).

The expressions of bile acid transport-related proteins, such as BSEP and MRP2, were detected by western blot (Figure 7A,B). The levels of BSEP and MRP2 in group M were remarkably downregulated in comparison with group C (*p* < 0.05), but they were all dramatically attenuated in the RCM extracts-treated groups (*p* < 0.05), particularly in the YH group.

## 4. Discussion

Chronic feeding of DDC has been studied as an in vivo model of cholestasis [7], which can cause liver injury and fibrosis. Prior to the formal experiment, we conducted a preliminary experiment to investigate the appropriate modeling dose of DDC for the induction of chronic cholestasis. Through the observation of liver morphology and the analyses of pathological sections, it was found that feeding 0.1% DDC to mice for 1 month was appropriate to establish the model of chronic cholestasis, which was consistent with the study of Zheng et al., [15]. Therefore, the modeling condition selected in this study was 0.1% DDC for 1 month. Current clinical drugs (such as UDCA) for cholestasis are limited by their therapeutic effects, while natural plant extracts have a higher safety profile compared with drug therapy. Numerous natural products with edible value have been developed as functional foods, including their active ingredients have also been reported to have hepatoprotective activity, and become adjuvant prevention or treatment of clinical diseases [26,27]. RCM fruits are widely cultivated as a condiment and beverage, and can also be used as a raw material to extract polyphenolic substances, such as gallic acid [28]. The extracts of RCM fruits have shown strong antioxidant activities in vivo and in vitro [21]. In this study, the preventive effect of the extracts of RCM fruits on cholestasis caused by DDC was investigated. Results exhibited that RCM had a good preventive effect on DDC-induced cholestasis, manifested as inhibiting inflammation and oxidative stress, promoting bile metabolism, and alleviating liver fibrosis. Overall, the fruits from RCM may be served as a potential natural material for functional foods to prevent and/or alleviate chronic cholestasis.

In this study, RCM fruit extracts could effectively alleviate liver injury caused by DDC. The levels of ALT and AST, as sensitive indicators of liver injury, were significantly increased in the plasma of DDC-treated mice. Generally, both of them are presented in the cytosol of liver cells and enter the bloodstream during liver injury [23]. The extracts of RCM had a significant inhibitory effect on the above adverse changes, indicating that the extracts of RCM fruits could ameliorate liver injury caused by DDC in mice. ALP is an enzyme that is widely distributed in the human liver and is discharged to the bile through the liver. It is a direct indicator to evaluate the level of biliary injury and the pathological process of cholestasis [4]. ALP will be produced in large quantities when bile cannot be excreted normally. The ALP activity of DDC-fed mice was obviously raised, which is characteristic of blocked bile excretion, while it was reversed by RCM extracts, suggesting that RCM could alleviate DDC-induced cholestasis. Similar to the finding that cholestasis is often accompanied by liver inflammation, DDC administration caused severe inflammatory cell infiltration and bile duct hyperplasia in liver tissue, however, these symptoms were improved after intervention with RCM extracts. Consistent with the previous study [15], the liver coefficient of the DDC group was also remarkably increased compared with RCM extracts-treated group.

Oxidative stress and inflammation are key mechanisms of DDC-induced cholestasis [29,30]. Bile duct hyperplasia and massive inflammatory cell infiltration were observed in DDC-treated mice. It has been reported that inflammatory cell infiltration can promote the bile duct epithelial cells to produce excessive reactive oxygen species (ROS), and then aggravate cell injury [31,32]. Antioxidant factors in the body, including GSH, CAT, and SOD, are the first stage of defense against excessive ROS production [28]. Meanwhile, the MDA level is a marker reflecting the degree of lipid peroxidation [23]. Increased oxidative stress factors in the liver in the cholestasis model suggested that oxidative stress is a crucial cause of cholestasis [4]. The extracts of RCM fruits can inhibit oxidative stress by increasing SOD, GSH, and CAT levels, and inhibit lipid peroxidation by reducing MDA content, which was consistent with the findings of Wei et al. [33] who reported that *herpetospermum pedunculosum* could attenuate cholestasis by increasing SOD, CAT, and GSH, and decreasing MDA. Oxidative stress can aggravate liver damage by acting on antioxidant factors. It can also stimulate macrophages to secrete inflammatory cytokines such as TNF-α, IL-6, and IL-1β, which further activate the NF-κB pathway [34]. Generally, IκBα acts as a dimer of NF-κB and inhibits NF-κB from entering the nucleus, causing the presence of NF-κB as an inactive substance in the cytoplasm. When inflammatory signals occur, IκBα is first phosphorylated and then degraded and separated from NF-κB. Isolated NF-κB phosphorylates into the nucleus, thus causing inflammation [20]. Activated NF-κB in turn promotes the release of inflammatory factors. A prior study reported that treatment with apigenin could attenuate DDC-induced cholestasis via adjusting the NF-κB pathway [15]. Similarly, the present study also suggested that the RCM fruit extracts could reduce the release of inflammatory cytokines and regulate the NF-κB pathway to prevent inflammatory responses.

Chronic cholestasis in DDC-fed mice eventually developed into fibrosis. Proliferating cholangiocytes can activate HSCs, which are the source of collagen deposition during fibrosis [35]. Biliary fibrosis induced by DDC is associated with excessive collagen deposition [36]. Masson staining and Sirius red staining, as common pathological methods, can be used to determine collagen deposition. Sirius red staining could also distinguish collagen types under polarized light, such as type I yellow and type II green [37]. Our results showed that collagen deposition occurred in the liver of DDC-fed mice, and RCM extracts significantly reduced the area of collagen deposition. DDC caused damage to intrahepatic small bile ducts, which subsequently proliferate and generate an inflammatory response, leading to the activation of HSCs [6]. Inflammatory cytokines have been researched to induce the activation of HSCs [38]. α-SMA is a marker of HSC activation [39]. Activated HSCs began to express α-SMA, and proliferated and differentiated into myofibroblasts secreting ECM [40]. The main component of extracellular matrix ECM is collagen, and its increase is the most direct way of hepatic fibrosis caused by the activation of HSCs [41]. A previous study showed that Huangqi decoction (HQD) could be used to treat DDC-induced cholestasis in mice by reducing the expression of TGF-β and α-SMA [42]. In the present study, compared with DDC-induced cholestasis mice, the expression of α-SMA in RCM extracts-treated mice was significantly decreased by IHC staining, which indicated that the extracts of RCM fruits could inhibit HSCs activation. The expression of TGF-β, a major fibrotic cytokine in fibrosis, was raised in chronic DDC-induced cholestasis. This study also indicated that the RCM fruit extracts could down-regulated the expression of TGF-β. In conclusion, the extracts of RCM fruits could inhibit the progression of fibrosis by reducing the expressions of TGF-β and α-SMA.

As a bile duct effector transporter, BSEP can transport cholic acid from hepatocytes to bile to prevent its deposition in the liver [43]. Once mice were fed with a DDC diet, the expression of BSEP would be restricted, which would further reduce bile acids and bile fluidity, causing cholestasis [44]. MRP2, a multidrug-resistant associated protein, is a specific organic anion transporter, which could control bile efflux [45]. The expression of MRP2 is significantly down-regulated in DDC-induced cholestasis, which leads to the accumulation of bile acids and aggravates cholestasis. In this study, it was found that the RCM extracts could up-regulate the expressions of proteins about bile acid transport, increase the bile acids efflux and reduce the bile acid deposition.

**Figure 8:**
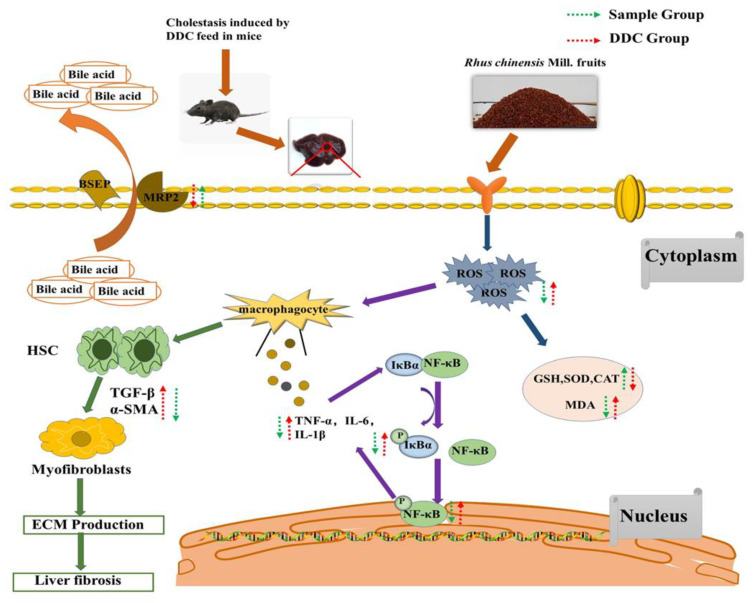
Schematic diagram of the main underlying mechanisms in DDC-induced cholestasis in mice with or without treatment with the RCM extracts.

In this paper, the preventive effect of RCM fruits on DDC-induced cholestasis and its possible mechanism was elucidated for the first time from the aspects of oxidative stress, inflammation, liver fibrosis, and bile acid transport (Figure 8). According to the result of UHPLC-HRMS/MS analysis (Table S1), phenolic compounds were the major secondary metabolites. Quantification results indicated that gallic acid and quercetin-3-O-rhamnoside are the most abundant phenolic compounds, accounting for about 49.15 and 23.32%, respectively, of the total identified phenolic compounds in the extract. In addition, two nonphenolic substances (citric acid and malic acid), which are organic acids common in many plants, were also observed. Thus far, no studies have reported the effects of these two organic acids on cholestasis. However, various studies have shown that many phenolic substances, such as apigenin, pterostilbene, and salvianolic acid B, have a preventive effect on cholestasis [15,46,47]. In the present study, the extracts of RCM contained different phenolic substances, which may also mainly contribute to the prevention of DDC-induced cholestasis in mice and their preventive effects might substantially depend on the phenolic compositions. Therefore, further studies are needed to investigate the phenolic compounds that play a major role in DDC-induced cholestasis, and the structure-activity relationships and synergies between these compounds, which will contribute to a better understanding and application of dietary phenolic substances to prevent DDC-induced cholestasis. Zhang et al. [48] studied the effect of ethanol extracts of RCM fruits on dextran sodium sulfate-induced colitis in C57 mice at a dose of 400 and 800 mg/kg b.w., the results showed that these two doses had no toxic effect on mice, and could exert good antioxidant and anti-inflammatory effects. Therefore, the selection of concentration in this paper was consistent with Zhang et al. Moreover, preliminary conversion [49] demonstrated that the intake of 400 and 800 mg/kg b.w. in mice in this study was approximately equivalent to the intake of 33.3 and 66.6 mg/kg b.w. in humans, respectively. Although both doses could be achieved in the daily diet, subsequent clinical trials are needed to determine the exact effective dose for daily intake in humans.

## 5. Conclusions

This paper demonstrated that administration of the RCM fruit extracts (especially at 800 mg/kg b.w.) could effectually prevent DDC-induced cholestasis. The RCM extracts markedly alleviated liver injury by inhibiting oxidative stress. Further analyses illustrated that the RCM extracts significantly attenuated inflammation by down-regulating the overactivation of the NF-κB pathway and the secretion of TNF-α, IL-1β, and IL-6. Moreover, the RCM extracts also attenuated cholestasis by modulating bile acid transporters and inhibiting liver fibrosis. Although further studies targeting a specific mechanism are needed to elucidate in subsequent experiments, the results of this study still clearly showed that RCM extracts could largely prevent DDC-induced cholestasis in mice. Therefore, it can be deduced that RCM fruits could be considered a potential dietary supplement to prevent and/or relieve chronic cholestasis.

## Data Availability

The data that support the findings of this study are available from the corresponding author upon reasonable request.

## Supplementary Materials

The following supporting information can be downloaded at: https://www.mdpi.com/article/10.3390/nu14194090/s1, Figure S1: The chromatograms of ethanol extracts of *Rhus chinensis* Mill. fruits.; Table S1: Phenolic compounds identified in *Rhus chinensis* Mill. fruits by UHPLC-ESI-HRMS/MS in negative mode.

## Abbreviations

UDCA	ursodeoxycholic acid
DDC	3,5-diethoxycarbonyl 1,4-dihydrocoridine
CAT	catalase
MDA	malondialdehyde
GSH	reduced glutathione
SOD	superoxide dismutase
H&E	hematoxylin-eosin
ROS	reactive oxygen species
IHC	immunohistochemical
SE	standard error
TNF-α	tumor necrosis factor-α
IL-6	interleukin-6
IL-1β	interleukin-1β
*p*-IκBα	phosphonated-inhibitor of nuclear factor kappaB
*p*-NF-κB	phosphorylated-nuclear factor κB
TGF-β	transforming growth factor-beta
α-SMA	α-Smooth muscle actin
BSEP	bile salt export pump
MRP2	multidrug resistance-associated protein 2
