# Supplementary material for: Protective Effect of Rhus chinensis Mill. Fruits on 3,5-Diethoxycarbonyl-1,4-Dihydrocollidine-Induced Cholestasis in Mice via Ameliorating Oxidative Stress and Inflammation
Source: Nutrients. 2022 Oct 1;14(19):4090. doi: 10.3390/nu14194090 (PMC9573408; doi:10.3390/nu14194090)
Supplement: Supplementary file 1 [file nutrients-14-04090-s001.zip › nutrients-1930590-supplementary.pdf]

Supporting Information

Characterization of the major bioactive compounds in the ethanol extract of *Rhus chinensis* Mill.

The major bioactive compounds in the ethanol extract of *Rhus chinensis* Mill. fruits were separated by using a Thermo Fisher Ultimate 3000 UHPLC System (Thermo Fisher Scientific, Germany) coupled with an Agilent Zorbax SB-C18 column (1.7 μm, 2.1 mm × 100 mm), and then characterized by a high-resolution mass spectrometer (Q-Exactive Orbitrap, Thermo Fisher Scientific, Bremen, Germany) in the negative mode. The parameters of HPLC were set as follows: mobile phases, 0.1% formic acid in water (A) and acetonitrile (B); flow rate, 0.1 mL/min; elution procedure, 0–2min, 5% B; 2–4min, 5%–20% B; 4–12min, 20%–30% B; 12–15min, 30%–50% B; 15–18min, 50%–5% B; 18–20min, 5% B; column temperature, 30°C; volume of sample injection, 2.0 μl. The following Mass parameters were used in the current work: full MS scan range, 50–1000 m/z; auxiliary gas flow, 9 L/min; sheath gas flow rate, 33 L/min; sweep gas, 4 L/min; S-lens RF level, 50%; spray voltage, 3.3 kV, capillary temperature, 330 °C; heater temperature, 360 °C.

Figure S1. The chromatograms of ethanol extracts of *Rhus chinensis* Mill. fruits. Peaks identification and their MS data are shown in Table S1. The base peak chromatogram is shown in Fig. S1.

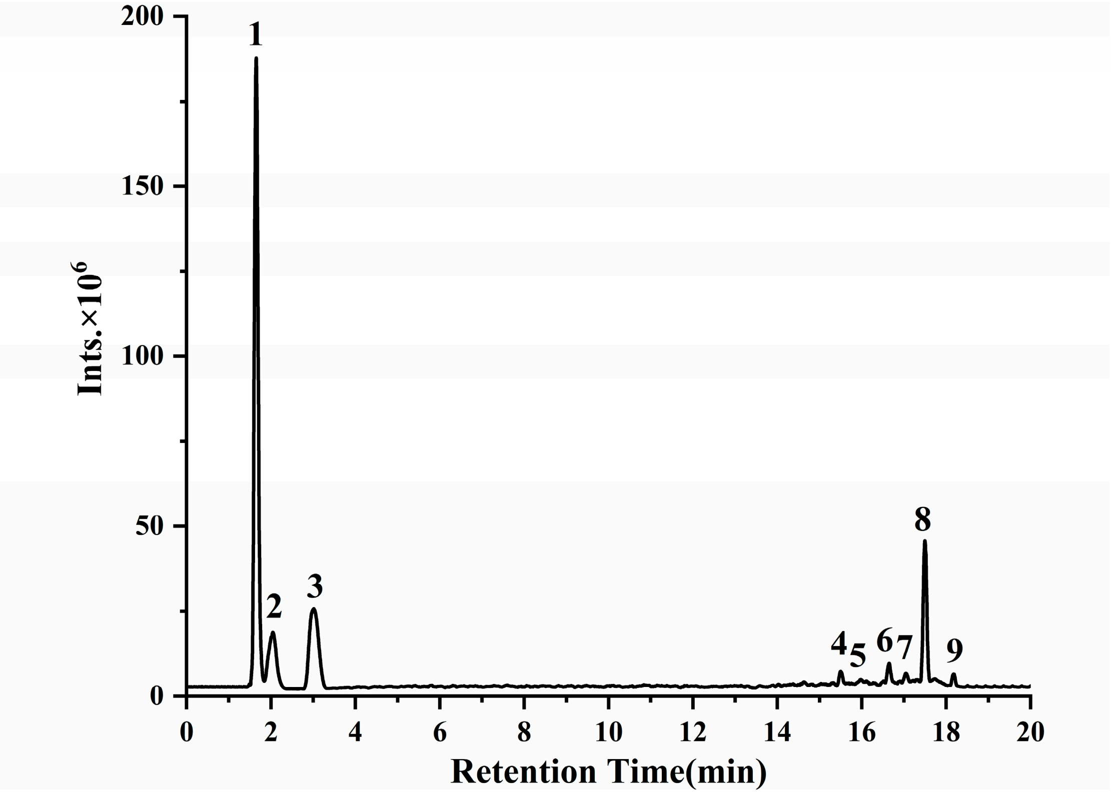

Table S1 Phenolic compounds identified in *Rhus chinensis* Mill. fruits by UHPLC-ESI-HRMS/MS in negative mode.

Peak No.	Compounds	Molecular formula	Retention time (min)	[M−H]$^{-}$ (*m/z*)	MS/MS ion fragments	Dry extract （μg/g）	Average percentage (%, Total identified phenolic content)
1	Malic acid	$C_4H_6O_5$	1.64	133.0130	71.0124(100), 72.9916(27.22)	---	---
2	Citric acid	$C_6H_8O_7$	2.14	191.0189	57.0332(100), 67.0174(39.17)	---	---
3	Gallic acid	$C_7H_6O_5$	3.01	169.0132	69.0331(100), 97.0281(52.73)	3892.57±165.42	49.15
4	Di-O-galloyl-glucoside	$C_{20}H_{20}O_{14}$	15.50	483.0779	300.0344(100),301.0344(83.27)	343.30±18.32	4.33
5	Trigalloyl glucose	$C_{27}H_{24}O_{18}$	15.99	635.0891	125.0234(100), 143.0331(12.55)	430.43±17.51	5.43
6	Myricetin-3-*O*-rhamnoside	$C_{21}H_{20}O_{12}$	16.64	463.0879	151.0025(100), 149.0234(89.09)	585.05±22.51	7.39
7	Myricetin-O-gallate	$C_{28}H_{24}O_{16}$	17.04	615.1002	151.0027(100), 137.0231(47.63)	595.55.±19.35	7.52
8	Quercetin-3-O-rhamnoside	$C_{21}H_{20}O_{11}$	17.49	447.0930	151.0026(100), 121.0283(15.48)	1847.03±116.85	23.32
9	Kaempferol-3-O-hexoside	$C_{21}H_{20}O_{10}$	18.17	431.0981	227.0347(100), 229.0544(77.81)	225.92±15.34	2.85

Values are expressed as the mean ± SD (n = 3, μg/g of dry extract); each standard curve was set with five different concentrations from 5.00 to 100.00 μg/mL depending on the response intensity. Gallic acid standard was used for quantifying the compounds 3,4 and 5; myricetin-3-*O*-rhamnoside standard was used for quantifying the compounds 6 and 7; quercetin-3-*O*-rhamnoside standard was used for quantifying the compounds 8; kaempferol standard was used for quantifying the compounds 9. All the phenolic standards were purchased from Must bio-technology CO., LTD (Chengdu, Sichuan, China) with purity ≤ 97%.
